# The role of bone marrow mesenchymal stromal cell derivatives in skin wound healing in diabetic mice

**DOI:** 10.1371/journal.pone.0177533

**Published:** 2017-06-08

**Authors:** Tomas de Mayo, Paulette Conget, Silvia Becerra-Bayona, Claudia L. Sossa, Virgilio Galvis, Martha L. Arango-Rodríguez

**Affiliations:** 1 School of Medicine Clínica Alemana Universidad del Desarrollo, Lo Barnechea, Santiago, Chile; 2 Center for Regenerative Medicine, School of Medicine Clínica Alemana Universidad del Desarrollo, Lo Barnechea, Santiago, Chile; 3 Universidad Autónoma de Bucaramanga (UNAB), Bucaramanga, Colombia; 4 Production Unity of Advanced Therapy, Fundación Ofalmológica de Santander, Clínica Carlos Ardila Lulle (FOSCAL Internacional), Bucaramanga, Colombia; 5 Centro Oftalmológico Virgilio Galvis, Bucaramanga, Colombia; 6 Fundación Oftalmológica de Santander FOSCAL, Bucaramanga, Colombia; Children's Hospital Boston, UNITED STATES

## Abstract

Mesenchymal stromal cells (MSCs) have shown to be a promising tool in cell therapies to treat different conditions. Several pre-clinical and clinical studies have proved that the transplantation of MSCs improves wound healing. Here, we compare the beneficial effects of mouse bone marrow-derived allogeneic MSCs (allo-mBM-MSCs) and their acelullar derivatives (allo-acd-mMSCs) on skin wound healing in Non-Obese Diabetic (NOD) mice. One dose of allo-mBM-MSCs (1×10^6^ cells) or one dose of allo-acd-mMSCs (1X) were intradermally injected around wounds in 8–10 week old female NOD mice. Wound healing was evaluated macroscopically (wound closure) every two days, and microscopically (reepithelialization, dermoepidermal junction, skin appendage regeneration, leukocyte infiltration, vascularization, granulation tissue formation, and density of collagen fibers in the dermis) after 16 days of MSC injection. In addition, we measured growth factors and specific proteins that were present in the allo-acd-mMSCs. Results showed significant differences in the wound healing kinetics of lesions that received allo-acd-mMSCs compared to lesions that received vehicle or allo-mBM-MSCs. In particular, mice treated with allo-acd-mMSCs reached significantly higher percentages of wound closure at day 4, 6 and 8, relative to the allo-mBM-MSCs and vehicle groups (p < 0.05), while wound closure percentages could not be statistically distinguished between the allo-mBM-MSCs and vehicle groups. Also, allo-acd-mMSCs had a greater influence in the skin would healing process. Specifically, they caused a less pronounced inflammatory severe response (p < 0.0001), more granulation tissue formation at an advanced stage (p < 0.0001), and higher density of collagen fibers (p < 0.05) compared to the other groups. Nevertheless, at day 16, both allo-mBM-MSCs and allo-acd-mMSCs revealed a higher effect on the recovery of the quality skin (continuous epidermis; regular dermoepidermal junction and skin appendages) relative to untreated lesions (p < 0.0001), but not between them. On the other hand, ELISA analyses indicated that the allo-acd-mMSCs contained growth factors and proteins relevant to wound healing such as IGF-1, KGF, HGF, VEGF, ANG-2, MMP-1, CoL-1 and PGE2. Compared to allo-acd-mMSCs, the administration of allo-mBM-MSCs is insufficient for wound healing in diabetic mice and delays the therapeutic effect, which maybe explained by the fact that trophic factors secreted by MSCs are critical for skin regeneration, and not the cells per se, suggesting that MSCs may require some time to secrete these factors after their administration.

## Introduction

Ideal healing of a skin wound requires an integration of complex biological and molecular events of cell migration and proliferation, extracellular matrix deposition, angiogenesis, and remodeling [1]. Impairment of any of such processes would lead to wound chronicity. Despite having numerous causes, wound chronicity is primarily associated with diabetes, atherosclerosis, venous/pressure ulcers, vasculitis, and trauma [2]. Given the increasing prevalence of chronic wounds worldwide, and their marked outcomes on patient morbidity and mortality (let alone amputations), it is crucial to consider adequate and effective interventions to treat such debilitating wounds [3].

Multipotent mesenchymal stromal cells, also referred to as mesenchymal stem cells (MSCs), are an outstanding tool for cell therapy applications, not only because of their multipotent nature, but also due to their ability to home and engraft into damaged tissues [4, 5], release trophic factors [6], promote neovascularization [7], manage oxidative stress [8] and trigger an anti-inflammatory response [9]. MSCs are procured from live donors [10] and can be both efficiently expanded *ex vivo* [11], and transplanted without previous conditioning of the patients as opposed to total bone marrow or hematopoietic stem cell transplantation [12].

Several pre-clinical and clinical studies have previously reported that autologous or allogeneic MSCs from different sources are safe and therapeutic in the treatment of chronic wounds [13], diabetic foot ulcers [14], pressure ulcers [15], burn injuries [16, 17], surgical wounds [18, 19], limb ischemia [20], and radiation burns [21]. These studies indicate that wounds treated with MSCs show a qualitative improvement in histological characteristics. Specifically, they report superior rete ridge architecture, multi-layered structure, improved dermal-epidermal junction and the formation of new skin appendage structures, such as hair follicles and sebaceous glands [22–26].

Even though the mechanisms by which MSCs ameliorate skin damage have been a subject of debate for years, two theories may currently explain MSC therapeutic effects: bioactive soluble factor production (growth factors, cytokines and specific proteins) or MSC differentiation into dermal and epidermal cells [27, 28]. Nevertheless, it is still controversial whether MSCs can contribute significantly to the regeneration of injured skin via tissue specific differentiation. For instance, Wu *et al*. used an excisional wound model in normal and diabetic mice to prove that only a small percentage of the MSCs that migrate to injury sites in response to chemotactic signals become incorporated and survive within wounded tissue [29]. Likewise, other studies have revealed that transplanted MSCs do not need to be close to the damaged site to promote wound repair and functional recovery [30].

Considering these previous findings, we compare the beneficial effects of mouse bone marrow-derived allogeneic MSCs (allo-mBM-MSCs) and their acelullar derivatives (allo-acd-mMSCs) to cutaneous wound healing in a full thickness excisional wound model in diabetic mice [31]. Allo-mBM-MSCs or their allo-acd-mMSCs were injected into Non-Obese Diabetic (NOD) mice. Wound healing was assessed macroscopically every two days in terms of wound closure, and microscopically after 16 days of the injection (reepithelialization, dermoepidermal junction, skin appendage regeneration, leukocyte infiltration, vascularization, granulation tissue formation, and density of collagen fibers in the dermis). The results revealed that although both treatments promoted tissue repair/regeneration, the allo-acd-mMSCs were the most effective tool for accelerating wound closure and the recovery of the damaged tissue. Based on ELISA analyses, the presence of relevant paracrine factors in allo-acd-mMSCs may play a significant role in the modulation of the wound healing process.

## Materials and methods

### Animals

C57BL/6 and NOD mice (Jackson Laboratory, Bar Harbor, ME) were used in the experiments. They were kept at constant temperature and humidity, with a 12:12 hour light-dark cycle and unrestricted access to standard diet and water. The animals were anesthetized with 1.1 mg/kg of 2,2,2-tribromoethanol (Avertin, Sigma-Aldrich) or sevofluorane (Abbott, Japan) when required. All animal procedures were approved by the Ethics Committee of the School of Medicine *Clínica Alemana—Universidad del Desarrollo School of Medicine* (approval ID:2011–14).

### Isolation and *ex vivo* expansion of allo-mBM-MSCs

Allo-mBM-MSCs were collected by flushing the femurs and tibias of 8-week-old C57BL/6 male mice with Alpha-MEM medium (Gibco, Auckland, NZ) followed by centrifugation. Cells were plated at a density of 1×10^6^ nucleated cells/cm^2^ and cultured in Alpha-MEM supplemented with 10% fetal bovine serum (HyClone Laboratories, Logan Utha) and 80 μg/mL gentamicin (Sanderson Laboratory, Chile), cultures were maintained at 37°C in 5% CO2 and 95% air atmospheric condition. After 72 hours, non-adherent cells were removed and fresh medium was added to the cells.

Adherent cells were detached after 96 hours using 0.25% trypsin and 2.65 mM EDTA (Life Technologies, Grand Island, NY), then centrifugated and sub-cultured at 7,000 cells/cm^2^. Allo-mBM-MSCs were injected after one subculture.

### Allo-acd-mMSCs preparation

The allo-acd-mMSCs were produced using allo-mBM-MSC cultures at 80% confluence (passage 1) in 10 cm tissue culture dishes (1×10^6^ cells approximately). Allo-mBM-MSCs were pre-washed twice with serum-free Alpha-MEM medium, maintained using 3 mL/dish of this medium and incubated for 24 hours under normoxia condition (37°C in a humidified atmosphere containing 95% air and 5% CO2). The medium was collected and centrifuged at 1500 rpm for 5 min. The supernatant was re-centrifuged at 3000 rpm for 3 min followed by collection of the second supernatant, named allo-acd-mMSCs to remove all cell debris. The pH was confirmed using an electric pH meter. Total allo-acd-mMSCs were collected, filtered, mixed, and aliquoted in 500 μL for storage at −20°C.

### Animal diabetic model: Induction and evaluation of diabetes

Diabetes was induced by a single intraperitoneal (i.p) injection of streptozotocin (STZ 200 mg/kg; CALBIOCHEM, San Diego, CA). For its activation, the STZ was prepared in citrate buffer solution (pH 4.5) immediately before its application. STZ was administered during the fasting state to pre-diabetic (8–10 week old) female NOD mice (body weight of 19–23 g). Hyperglycaemia usually occurred 2 days after STZ injection, and was verified using blood collected from the tail vein with a portable glucose meter (Accu-Check Go; Roche, Nutley, NJ, USA). Mice with blood sugar levels greater than or equal to 250 mg/dL after two consecutive measurements were considered diabetic. Once hyperglycaemia was confirmed, diabetic mice were randomly assigned to receive one of the following treatment options: allo-mBM-MSCs, allo acd-mMSCs or vehicle (saline solution with 5% autologous plasma). During the study, glucose levels were measured every four days.

### Excisional skin wound model

One week after administration of STZ, 8–10 week old female NOD mice were anesthetized with avertin and the hair was removed from the dorsal surface. Excisional biopsy wounds were made using a 6 mm punch on one side of the midline extending through the panniculus carnosus as previously described by Galiano *et al*. [31]. The number of mice used for each condition was: 25, 27 and 25 for the allo-mBM-MSC, the allo-acd-mMSC and the vehicle treatment, respectively.

### MSC and acellular derivative injection

Allo-mBM-MSCs derived from 8-week-old C57BL/6 male mice (1x10^6^ cells in 60 μL of vehicle) were injected intradermally in the periphery of the wound at four sites. Similarly, one dose (1X) of allo-acd-mMSCs (100 μL) or vehicle (60 μL) was intradermally injected around the wound. After the injections, tegaderm (3M, London, ON, Canada) was immediately placed over the wounds. Animals were housed individually during the experiment.

### Evaluation of the wound healing process

Wound size was measured in duplicate using a digital caliper (Mitutoyo Sul Americano LTDA, Brazil) every two days after the allo-mBM-MSC or allo-acd-mMSC administration to compute the wound areas. Also, digital photographs of the wounds were taken (FUJIFILM-Finepix HS20 EXR). Time elapsed to wound closure was defined as the time in which the wound bed became completely reepithelialized and filled with new tissue. The percentage of wound closure was calculated using the formula [(area of original wound – area of actual wound)/(area of original wound)] x 100. All wounds were closed after 16 days and the mice were sacrificed by an overdose of anesthesia (ketamine 50 mg/kg – xylazine 5 mg/kg, Centrovet). Skin samples from the affected area and 2 mm of the surrounding skin were obtained using an 8 mm biopsy punch.

### Histological analyses

Harvested skin samples from euthanized mice were fixed in 10% formalin, and embedded in paraffin. Four *μ*m thick skin sections were cut using a Leica RM 2125 RTS microtome (Wetzlar, Germany) and stained with haematoxylin-eosin (Sigma-Aldrich) and Masson’s trichrome (Diagnostic Biosystems, Pleasanton, CA). Computational analysis was performed to quantitatively assess dermo-epidermal junction integrity. In brief, the border of the region with incomplete junction was manually selected using the open access software ImageJ, which calculated the traced area in pixels. Depending on this area, a score was assigned: 0: >2,801 pixels (corresponding to injunction); I: 1,201–2,800 pixels; II: 751–1,200 pixels; III: 401–750 pixels; and IV: 0.1–400 pixels (corresponding to complete junction) (S1 Fig).

A qualified pathologist standardized valid histological criteria in regular haematoxylin-eosin staining to estimate the degree of leukocyte infiltration (unquantifiable subjective parameters). For these purposes, an estimation of the area occupied by inflammatory cells (lymphocytes, plasma cells, eosinophils, macrophages, neutrophils, etc.) was determined in the superficial or mild/deep dermis. The following scale was used: absent (no apparent inflammatory response); mild (< 10% of the area covered by inflammatory cells); moderate (10 to 50% of the area covered by inflammatory cells) and severe (> 50% of the area covered by inflammatory cells).

In addition, a classification of granulation tissue stage (young or advanced) was assessed based on the degree of the fibroblastic response, vascular proliferation, correlation with the inflammatory response, and gross morphological evaluation of collagen fibers.

Moreover, appendage-like structures in each wound section were studied using the Masson’s trichrome staining. Also, morphometric computational analysis of dermal collagen was conducted with Masson’s trichrome staining to provide quantitative analysis of the density and the intensity of dermal collagen fibers according to the method described by Miot and Brianezi [32].

On the other hand, keratinocytes were stained with a monoclonal antibody against epidermal keratin subunits (Abcam Inc, Cambridge, MA) and visualized with a secondary antibody Alexa Fluor 488 (Abcam Inc, Cambridge, MA, USA). Similarly, vascularization was identified with polyclonal Von Willebrand Factor antibody (Dako, Denmark), followed by incubation with secondary antibody Alexa Fluor 488 (Cell Signaling, Massachusetts, USA). Nuclear staining was performed with DAPI (AppliChem, Germany). All slides were examined under a Leica DM2000 microscope (Wetzlar, Germany), and images were captured with a Leica DFC 295 camera (Wetzlar, Germany).

### ELISA assays

The allo-acd-mMSCs samples were evaluated for their content of specific growth factors and proteins relevant to wound healing via ELISAs. Specifically, the following factors were measured per manufacturer’s instructions: collagen type 1 (CoL-1), keratinocyte growth factor (KGF), matrix metalloproteinase 1 (MPP-1), matrix metalloproteinase 3 (MPP-3), angiopoietin 1 (Ang-1), angiopoietin 2 (Ang-2), human insulin-like growth factor 1 (IGF-1), hepatocyte growth factor (HGF), vascular endothelial growth factor (VEGF), prostaglandin E2 (PGE_2_) and epidermal growth factor (EGF) (kits from R&D Systems, Abcam and MyBioSource).

### MSC Characterization

#### Colony forming unit (CFU) assay

Allo-mBM-MSCs cells were suspended in alpha-MEM supplemented and plated at 17,000 nucleated cells/cm^2^ density in triplicate. Non-adherent cells were removed with fresh medium, which was changed twice a week. Cells were stained with 0.5% crystal violet (Sigma-Aldrich, St. Louis, MO) in 10% methanol for 20 minutes on day 21. The colonies so formed were counted after four washes and the results were expressed as CFU/million nucleated cells plated (S2A Fig).

#### Proliferation assay

First passaged allo-mBM-MSCs were sub-cultured at 4,000 cells/cm^2^; the medium was changed every three days. The amount of cells was determined on days 0, 3, 6, 9 and 12 after staining with 0.5% crystal violet in 10% methanol for 20 minutes. Cell-incorporated crystal violet was solubilized after four washes by incubation with phosphate buffer in methanol (50:50) and spectrophotometrically quantified (570 nm absorbance) (S2B Fig).

#### MSC differentiation assay

Allo-mBM-MSCs were cultured either in osteogenic (0.1 μM dexamethasone, 10 mM β- glycerophosphate, 50 μg/ml ascorbic acid [33] or adipogenic (1μM dexamethasone, 100 μg/ml 3-isobutyl- 1-methylxanthine (IBMX) medium [34] medium for 21days, respectively (S2C Fig).

#### Immunophenotyping

Although there is no current consensus with respect to murine MSCs markers (versus those for human MSCs), immunophenotyping was performed by flow cytometry analysis. In brief, cells were incubated with anti-CD45.2 clone 104 (APC-eFluor780 conjugated, eBioscience), anti-CD11b, clone M1/70 (PE-Cy-conjugated, eBioscience), anti-Sca-1, clone D7 (PE-conjugated, eBioscience), anti-CD90.2 clone 53–2.1 (PE-Cy7-conjugated, BD Pharmingen^™^) and anti-ASMA, clone 1A4 (FITC-conjugated, Sigma) (S2D–S2F Fig).

### Statistical analysis

Stat Graph Prism 5.0 software was used for statistical analysis. Data are reported as the mean ± standard error of the mean. Comparison of experimental groups was performed using ANOVA followed by Dunn’s multiple test, p < 0.05. The association or independence of categorical variables were compared using Pearson´s chi–square test, a p < 0.0001 values was accepted as statistically significant.

## Results

### Wound closure kinetics in diabetic mice

**Fig 1:**
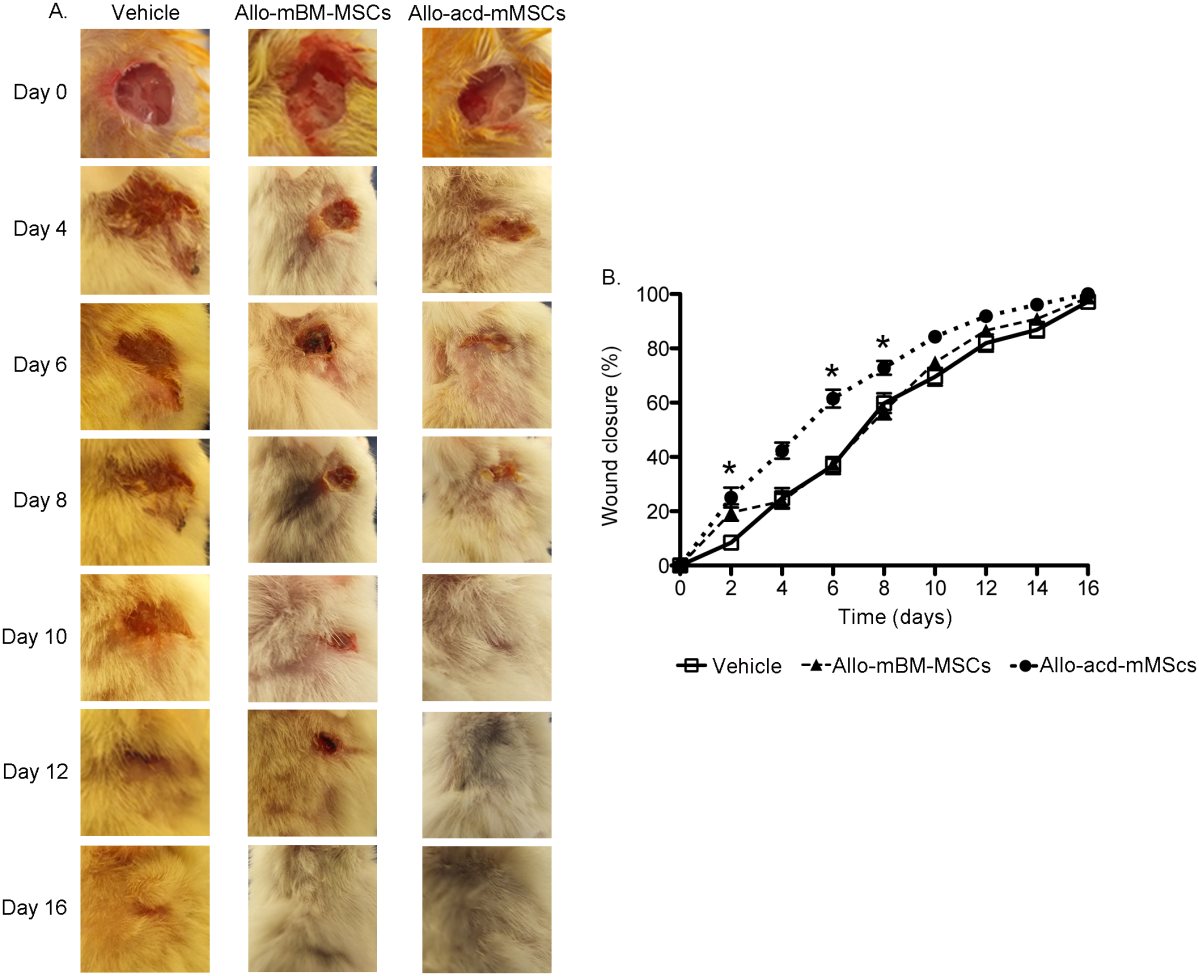
Effects of allo-mBM-MSCs or allo-acd-mMSCs on wound closure in murine excisional wounds. (**A)** Representative images of the wounds in NOD mice under vehicle, allo-mBM-MSCs or allo-acd-mMSCs treatments at day 0 (before treatment), 4, 8, 10, 12 and 16 for NOD wounds. (**B)** Wound closure analysis. All wounds were measured using digital calipers at day 0, 2, 4, 6, 8, 10, 12 and 16 post-operation. The wound closure rate is plotted as the reduction percentage of original wound area over time (n = 25, 25 and 27 for vehicle, allo-mBM-MSCs and allo-acd-mMSCs groups, respectively). Analysis of variance (ANOVA) was used, * indicates a significant difference (p < 0.05). Abbreviations: NOD, Non-Obese Diabetic; allo-mBM-MSCs, mouse bone marrow-derived allogeneic MSCs and allo-acd-mMSCs, mouse bone marrow acelullar derivatives allogeneic MSCs.

In order to determine whether the paracrine effect of allo-acd-mMSCs was sufficient in the wound healing process, allo-acd-mMSCs and allo-mBM-MSCs were injected into excisional wounds in NOD mice. As shown in Fig 1A, wound closure started to be noticed after 4 days for the allo-acd-mMSCs treated mice, and became more evident after 8 days compared to the allo-mBM-MSCs and vehicle counterparts (4 *vs* 6 days, respectively). In addition, wound closure percentage was significantly higher in the allo-acd-mMSC treated mice, relative to the other two groups (Fig 1B). Specifically, mice treated with allo-acd-mMSCs reached significantly higher percentages of wound closure at days 4, 6 and 8, relative to the allo-mBM-MSCs and vehicle groups (p *<* 0.05), while wound closure percentages between the allo-mBM-MSCs and vehicle samples could not be statistically distinguished. Moreover, the data suggested that 50% of wound closure was reached approximately after 5 days for the allo-acd-mMSCs treated mice, whereas for the other two conditions the same extent of wound closure was achieved after approximately 7 days of treatment. Cumulatively, the results revealed that allo-acd-mMSCs were the most effective tool for wound closure.

### Recovery of regenerated skin in diabetic mice

**Fig 2:**
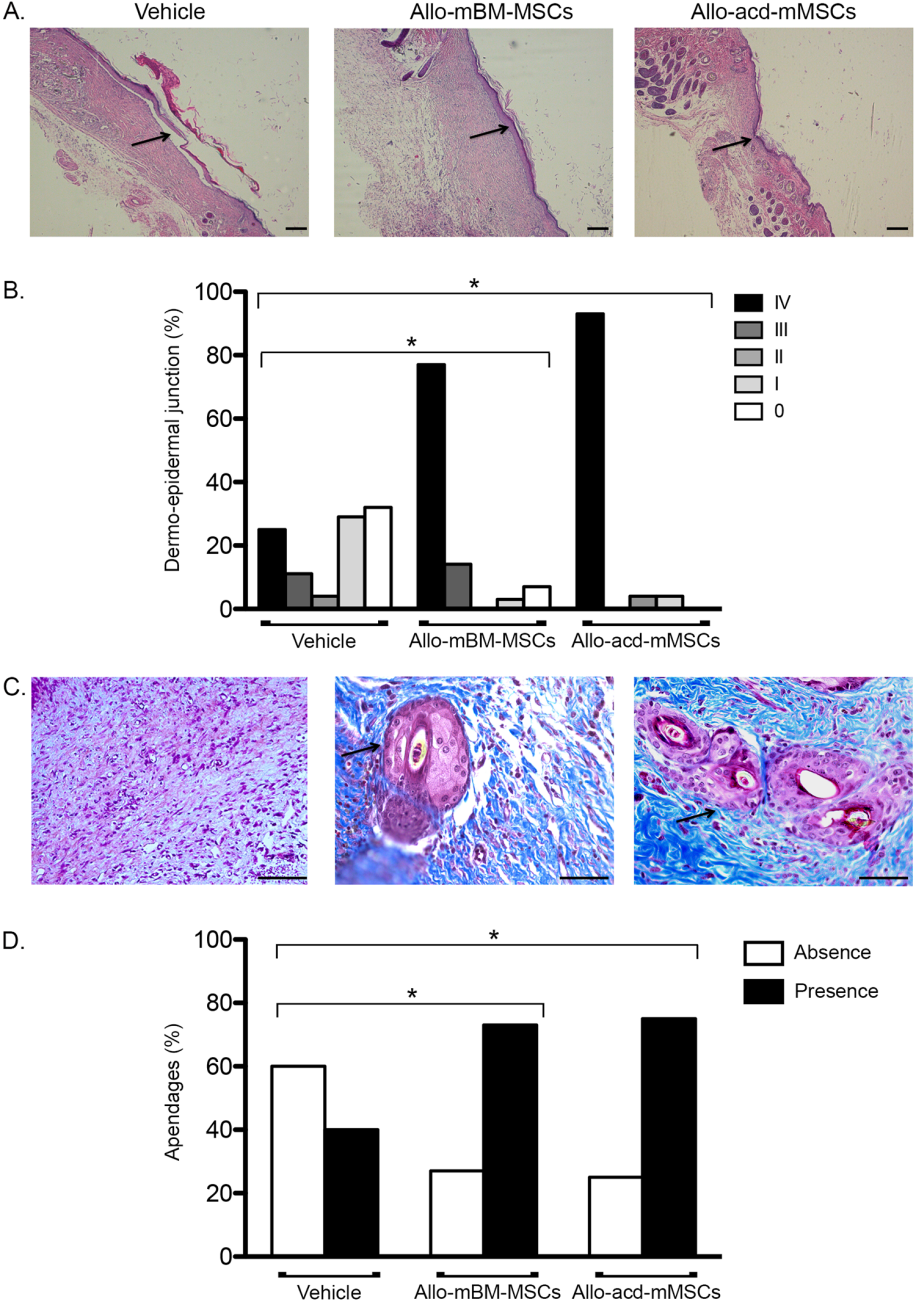
Histological analysis of dermo-epidermal junction and appendage-like structure formation of skin wounds 16 days after treatment with allo-mBM-MSCs and allo-acd-mMSCs in NOD mice. (A) Wound histological representative images of haematoxylin-eosin staining showing dermo-epidermal junction, which are indicated by arrows. Scale bar = 100 *μ*m. (B) Frequency of animals with the histological scores for dermo-epidermal junction. The score was assigned based on the junction level and was quantified using ImageJ (scores: IV: 0.1–400 pixels (complete junction), III: 401–750 pixels, II: 751–1,200 pixels, I: 1,201–2,800 pixels and 0: > 2,801 (incomplete junction). (C) Representative images of Masson’s trichrome staining illustrating appendage-like structure in the dermis indicated by arrows. Scale bar = 50 *μ*m. (D) Frequency of animals with the score for appendage-like structure. (n = 25, 25 and 27 for vehicle, allo-mBM-MSCs and allo-acd-mMSCs groups, respectively). Analysis of Pearson’s chi-square test was used, * indicates a significant difference (p < 0.0001). Abbreviations: NOD, Non-Obese Diabetic; allo-mBM-MSCs, mouse bone marrow-derived allogeneic MSCs; allo-acd-mMSCs and mouse bone marrow acelullar derivatives allogeneic MSCs.

In order to determine the quality of the newly-formed skin, reconstitution of the dermo-epidermal junction, new skin appendage structure and reepithelialization at day 16 were evaluated. Significant differences in the complete reconstitution of the dermo-epidermal junction were identified in mice treated with allo-mBM-MSCs and allo-acd-mMSCs (score IV) compared to animals treated with vehicle (p < 0.001) (Fig 2A and 2B). Likewise, new skin appendage structure (hair follicles or sebaceous glands) were detected in the wounds treated with allo-acd-mMSCs and allo-mBM-MSCs compared to wounds treated with vehicle (p < 0.001) (Fig 2C and 2D).

Reepithelialization were observed between cutaneous lesions treated with the vehicle and the ones which received either allo-acd-mMSCs or allo-mBM-MSCs, the wounds showed the formation of normal layers of the epithelium as demonstrated by the expression of pancytokeratin, an epithelial tissue marker (S3A Fig).

### Skin healing process in diabetic mice

**Fig 3:**
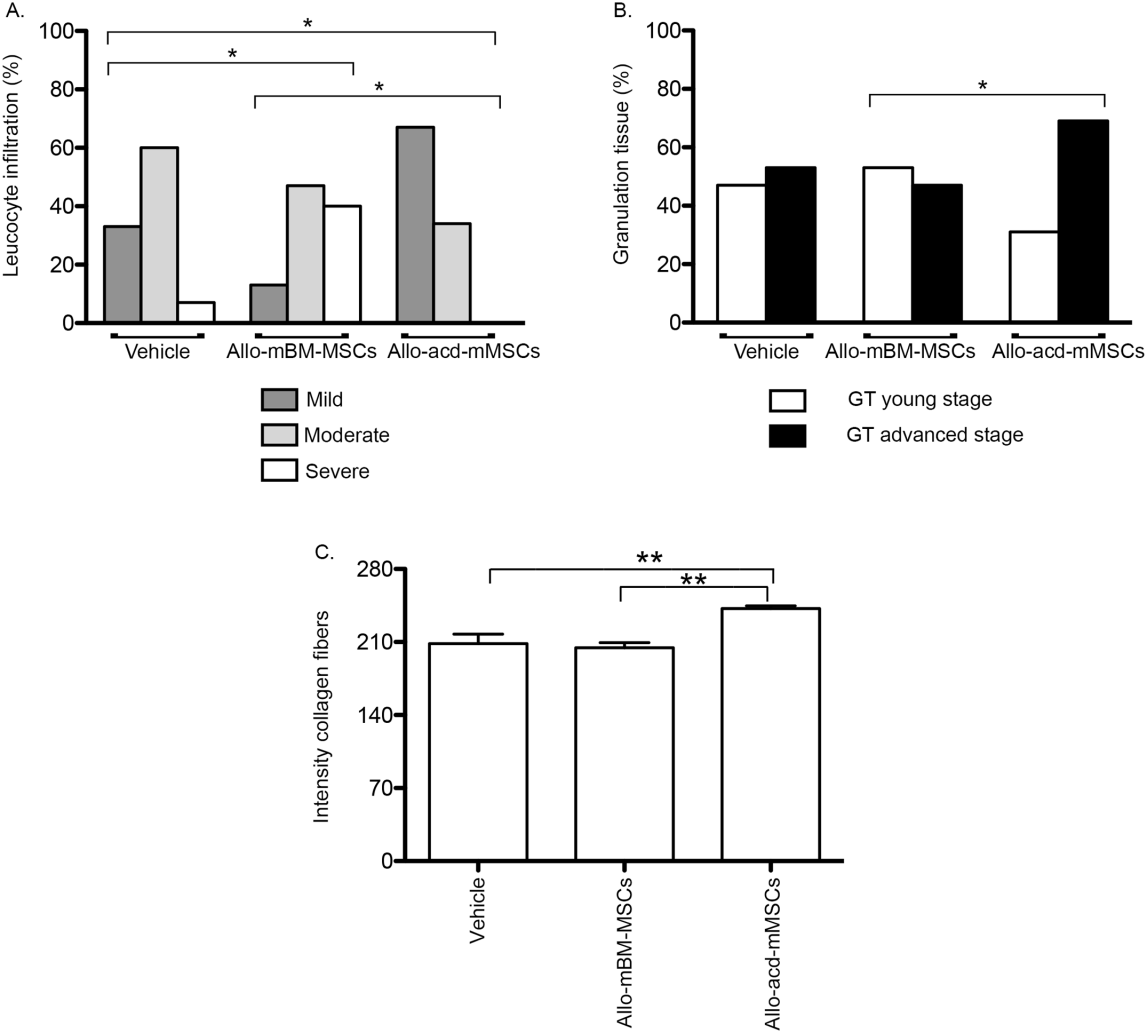
Histological analysis of leucocyte infiltration, granulation tissue and collagen fibers of skin wounds 16 days after treatment with allo-mBM-MSCs and allo-acd-mMSCs in NOD mice. Frequency of animals with the histological scores for: **(A)** leukocyte infiltration in the dermis, (**B**) granulation tissue formation stage and (**C**) quantitative analysis of the intensity of dermal collagen fibers. (n = 25, 25 and 27 for vehicle, allo-mBM-MSCs and allo-acd-mMSCs groups, respectively). Pearson’s chi-square test and variance (ANOVA) analysis were used, * indicates a significant difference p < 0.0001 and ** p < 0.05. Abbreviations: NOD, Non-Obese Diabetic; allo-mBM-MSCs, mouse bone marrow-derived allogeneic MSCs and allo-acd-mMSCs, mouse bone marrow acelullar derivatives allogeneic MSCs.

Sixteen days after the start of the treatment, the influence of allo-acd-mMSCs on wound healing was evaluated by estimating: i) the magnitude of inflammatory infiltrates, ii) granulation tissue formation (including its stages), iii) the density of the collagen fibers in the cutaneous lesion and iv) vascularization. As shown in Fig 3A, more severe leucocyte infiltration was found in the dermal connective tissue of the allo-mBM-MSC and vehicle treated groups compared to the group treated with allo-acd-mMSCs (p < 0.001).

Comparison of the extent of granulation tissue formation in the dermal connective tissue indicated higher granulation tissue in advanced stage in most of the animals treated with allo-acd-mMSCs relative to the animals treated with allo-mBM-MSCs (p < 0.001) (Fig 3B). Moreover, young-stage granulation tissue formation was characterized by distinct histopathological changes such as an acute inflammatory process with mild to moderate interstitial edema, the presence of collagen fiber deposits, and the fibroblastic response. On the contrary, granulation tissue formed at an advanced stage was identified by a scarce inflammatory infiltrate, less pronounced fibroblastic reaction, high presence of dense collagen fiber deposition and emerging hyalinization, which was sometimes admixed with hemosiderin-laden macrophages.

In the same way, a higher density and intensity of collagen fibers were found in the allo-acd-mMSCs-treated group (mean 242) compared to the allo-mBM-MSC (mean 204) and vehicle (mean 208) groups (p < 0.05, Fig 3C). These data suggested that a more intense remodeling process takes place in the allo-acd-mMSCs samples, compared to its allo-mBM-MSCs and vehicle counterparts, in which the collagen fibers were less dense and organized. In addition, allo-acd-mMSCs showed greater vascular proliferation compared to allo-mBM-MSCs or vehicle treated wounds (S3B Fig).

### Profile of paracrine factors found in allo-acd-mMSCs

**Fig 4:**
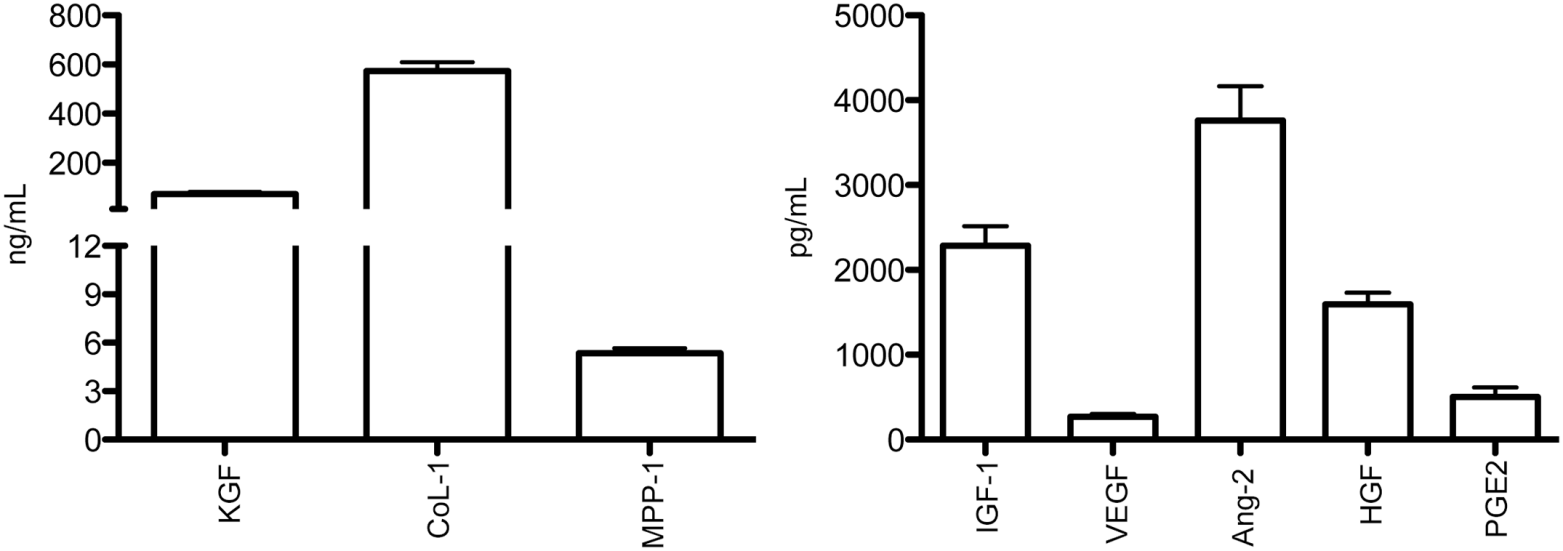
Growth factors and protein profile relevant to wound healing found in allo-acd-mMSCs. ELISA measurement of secretion levels of KGF, CoL-1, MPP-1, VEGF, IGF-1, Ang-2, HGF, and PGE_2_ were measured after 24 h allo-mBM-MSCs culture. (n = 24). Abbreviations: allo-mBM-MSCs, mouse bone marrow-derived allogeneic MSCs; allo-acd-mMSCs, mouse bone marrow acelullar derivatives allogeneic MSCs, KGF, keratinocyte growth factor; CoL-1, collagen type 1; MPP-1, matrix metalloproteinase 1; VEGF, vascular endothelial growth factor; Ang-1, angiopoietin 1; IGF-1, human insulin-like growth factor 1; Ang-2, angiopoietin 2; HGF, hepatocyte growth factor and PGE_2_, prostaglandin E2.

Paracrine factors secreted by MSCs play a crucial role in wound healing [25, 35–38]. Therefore, the levels of growth factors and specific proteins relevant to wound healing were quantified to gain insight into the relationship between paracrine factor concentration and the observed response in terms of wound closure kinetics, characteristics of the regenerated skin and the healing process. As illustrated in Fig 4, the growth factors and proteins with the highest concentration in the allo-acd-mMSCs were CoL-1, KGF, MPP-1 and Ang-2. In contrast, IGF-1, HGF, PGE2 and VEGF were present in lower concentrations. Finally, EGF, Ang-1 and MPP-3 were not detected in the allo-acd-mMSCs.

## Discussion

The present study was designed to examine the impact of allo-acd-mMSCs relative to allo-mBM-MSCs on diabetic wound healing toward the application of novel therapeutic approach for this condition. The results demonstrated that allo-acd-mMSCs possess more robust therapeutic properties than allo-mBM-MSCs *per se* for wound healing in diabetic mice. Particularly, the current work revealed that injection of allo-acd-mMSCs accelerated wound closure, resulting in improved healing, relative to allo-mBM-MSC injection. These results are consistent with previous studies that suggest that paracrine factors play a key role in MSC contribution to skin wound healing [22, 25, 35–41]. Nevertheless, our findings are in contrast to the results obtained by Wu *et al*., who demonstrated that the beneficial effect of MSCs on wound healing in nondiabetic and diabetic mice is primarily achieved through cell differentiation, proposing a direct contribution of MSCs to cutaneous regeneration [29]. Despite this fact, the literature evidences that the contribution of MSC differentiation to wound healing is limited because of poor engraftment and survival of MSCs at the injured site [38, 42].

Significant improvement regarding the functional and histological quality of healed skin, such as superior rete ridge architecture, multilayered structure and major dermoepidermal junction, was detected in a greater degree in the groups treated with allo-mBM-MSCs and allo-acd-mMSCs, relative to the control group (vehicle). These results are consistent with other studies that have demonstrated that wound reepithelialization is the main mechanism contributing to wound closure in the presence of BM-MSCs or BM-progenitors cells [43, 44]. In addition, allo-mBM-MSCs or their allo-acd-mMSCs stimulated the formation of new skin appendage structure, for instance, hair follicles or sebaceous glands, characteristics that are considered signs of skin regeneration. This potential benefit has also been reported by other authors who used BM-MSCs, human umbilical cord derived MSCs [45], or products derived of these cells such as exosomes [46] or conditioned medium [29].

Historically, MSCs have been considered hypoimmunogenic because they exhibit low expression levels of MHC class I markers, no expression of MHC class II markers, and no expression of costimulatory molecules, which allow them to avoid immunosurveillance [5, 47, 48]. In addition, it is known that MSCs have an immunoregulatory effect given by different putative mechanisms including immune cell interaction, production of soluble factors and promoting of T-reg generation [49]. However, in the present work, large severe leukocyte infiltration in wounds that received allo-mBM-MSCs was found, relative to the allo-acd-MSCs group, after 16 days of treatment. These results are in agreement with some studies that have previously reported concerns related to the immunogenicity of transplanted allogeneic MSCs [5, 48, 50]. MSCs have been correlated to not be intrinsically immunoprivileged, as assumed earlier [51]. Specifically, some reports indicated that allogenic MSCs can up-regulate the expression of MHC II and costimulatory molecules under certain conditions such as in the presence of inflammatory milieu through the IFN-γ stimulation [5]. MSCs can be lysed by activated NK cells by the interaction of NK receptors (NKp30, NHG2D and DNAM-1) with the MSCs ligands [5, 52], induce memory T cells [53] and trigger the formation of IgG antibodies after subcutaneous or intracardial injection, which results in the rejection of allogeneic MSC grafts [54]. On the other hand, other studies have shown that the presence of soluble factors derived of MSCs such as TGF-β, HGF and PGE2, are capable of suppressing T-cell responses *in vitro* [5]. In this context, the last two factors were found in allo-acd-MSCs, suggesting that the absence of severe leukocyte infiltration in wounds that received allo-acd-MSCs, could be due to soluble factors of this type.

The formation of new blood vessels (neovascularization) is considered one of the early essential processes in wound healing to sustain both the newly formed granulation tissue and the survival of keratinocytes and re-epithelialization [44, 55]. Based on macroscopic evaluation, the extent of granulation tissue formation in the bed of the wounds at day 4 was higher in the allo-mBM-MSCs and allo-acd-mMSCs groups compared to the vehicle (S4 Fig).

Likewise, from the histological assessments, the experimental group treated with allo-acd-mMSCs showed greater vascular proliferation and granulation tissue in advanced stage relative to the groups treated with allo-mBM-MSCs or vehicle at day 16. For the latter groups, the granulation tissue was in a young phase with abundant proliferation fibroblastic type. This observation is consistent with one of the reported therapeutic functions of MSCs in wound healing where the early induction of granulation tissue followed by neovascular network formation leads to a fast healing process [55].

Another aspect to take into account about the quality of healed skin is the restoration of its dermal mechanical properties, which largely depend on the specific arrangement of collagen fibers [40]. In this context, non-healing wounds are associated with atypical matrix deposition that may be related to the overproduction or insufficient presence of growth factors [56]. The results of the present work demonstrated that wounds receiving allo-mBM-MSCs or vehicle had reduced density and organization of collagenous fibers at the dermal matrix compared to allo-acd-mMSC treated wounds. In particular, collagen fibers in the allo-mBM-MSC and vehicle treated wounds still appeared relatively thin and were parallel-oriented to the skin, which indicated improper wound maturation. On the contrary, allo-acd-mMSC treated wounds had thicker collagen fibers, distributed in a network-like manner mimicking normal skin.

Furthermore, the success of the wound healing process depends on a regulated secretion of growth factors, cytokines and chemokines that are involved in a complex integration of signals that coordinate cellular processes [57]. Non-healing wounds have been associated with the overproduction (acute wound) or insufficient presence (chronic wounds) of growth factors such as EGF, IGF-1, FGF-2, PDGF-BB, VEGF, Ang-1, SDF-1, KGF, MMP-9, or cytokines such as TGF-β, IL-1, IL-6, IL-8, and TNF-alpha [22, 56, 58]. These molecules could contribute towards wound repair and skin regeneration by suppressing inflammation, angiogenesis and stimulating skin stem cell proliferation and differentiation into new keratinocytes [23, 29, 55, 59–61]. We examined the presence of several of these growth factors in the allo-acd-mMSCs, and found a set of key bioactive molecules implicated in the wound healing process, namely, IGF-1, KGF, HGF, VEGF, Ang-2, CoL-1, MMP-1 and PGE2.

The pro-angiogenic growth factors IGF-1, Ang-2 had the highest concentration in the allo-acd-MSCs compared to VEGF. According to the literature, these factors have been reported to possibly enhance endothelial cell proliferation and neovascularization, a critical stage in tissue regeneration [62, 63]. In the same way, Col-1, which is found in the extracellular matrix, has been reported as crucial element in the restoration of the dermal mechanical properties and in a better remodeling of dermal fibrous architecture [40]. Our ELISA results showed high levels of this protein in the allo-acd-mMSCs.

## Conclusions

Cumulatively, our study demonstrated that administration of allo-acd-mMSCs is more effective in wound healing and cutaneous regeneration than allo-mBM-MSCs or vehicle in diabetic mice. The results revealed that allo-acd-mMSCs accelerated the kinetics of wound closure, reduced severe leukocyte infiltration, increased granulation tissue formation, and remodelled the collagen deposition orientation. Also, our data suggested that the presence of important bioactive molecules in the allo-acd-mMSCs could have initiated the wound healing mechanism and facilitated the host response to tissue repair. Allo-acd-MSCs might serve as a novel therapeutic approach to treat chronic wounds caused by pathological conditions or other trauma.

## Supporting information

S1 Fig Histological scores of dermo-epidermal junction integrity. Representative images of haematoxylin-eosin staining of the dermo-epidermal junction integrity. Image J was used for estimating unbound area size. Scores: IV: 0.1–400 pixels (complete junction), III: 401–750 pixels, II: 751–1,200 pixels, I: 1,201–2,800 pixels and 0: > 2,801 (incomplete junction). Scale bar = 100 μm and applied to all images.
(TIF)

S2 Fig Characterization of isolated mBM-MSCs from C57BL/6 mice. **(A)** Bone marrow abundance was determined by CFU assay. Data are mean ± s.e.m (n = 6). **(B)** Proliferation kinetics was evaluated by crystal violet staining (570 nm absorbance) over a period of 12 days. Data are mean ± s.e.m (n = 4). **(C)** mBM-MSCs differentiate to mesodermal lineages *in vitro*. Passage 2 mBM-MSCs were used for potential differentiation. Adipogenic differentiation: cells were cultured in adipogenic differentiation medium for 21 days. Oil Red O staining was performed to detect lipid accumulation. Osteogenic differentiation: cells were cultured in osteogenic differentiation medium for 21 days. Alizarin Red staining was performed to detect calcium accumulation. Scale bar 100 μm. **(D)** Flow cytometry analysis of mBM-MSC surface markers. mBM-MSCs expressed anti-CD90.2, anti-Sca-1, and anti-ASMA but not anti-CD45.2 and anti-CD11b. Representative images for 2 animals per group. **(E)** Statistical data of flow cytometry. Abbreviations: mBM-MSCs, mouse bone marrow-derived MSC and CFU, colony formation unit.
(TIF)

S3 Fig Effects of allo-mBM-MSCs and allo-acd-MSCs on reepithelialization and wound vascularity. Representative images of cutaneous wound sections on day 16 in NOD mice treated with vehicle, allo-mBM-MSCs and allo-acd-mMSCs were immunostained with **(A)** an anti- pan-cytokeratin antibody (green) and **(B)** an anti-von Willebrand Factor (vWF) positive blood vessels (green), both primary antibodies were probed with Alexa Fluo488 secondary antibody and nuclei (blue) were counterstained with 4-6-diamidino-2-phenylindole (DAPI). Representative results of 4 animals per experimental groups. Scale bar 50 μm. Abbreviations: NOD, Non-Obese Diabetic; allo-mBM-MSCs, mouse bone marrow-derived allogeneic MSCs; allo-acd-mMSCs, mouse bone marrow acelullar derivatives allogeneic MSCs; vWF, von Willebrand Factor; DAPI 4-6-diamidino-2-phenylindole.
(TIF)

S4 Fig Effects of allo-mBM-MSCs and allo-acd-MSCs on early granulation tissue formation on wound in NOD mice. Invasion of granulation tissue is shown from the wound margins into the wound bed after 4 days of treatment. Allo-mBM-MSCs and allo-acd-mMSCs groups showed higher presence of tissue with the following features: wet, reddish, soft and granular in gross appearance compared to the vehicle group (only sparse granulation tissue was observed). Scale bar = 50 μm (applied to all images). Abbreviations: allo-mBM-MSCs, mouse bone marrow-derived allogeneic; acelullar derivatives, allo-acd-mMSCs.
(TIF)
