# Supplementary figures and images for: The role of bone marrow mesenchymal stromal cell derivatives in skin wound healing in diabetic mice
Source: PLoS One. 2017 Jun 8;12(6):e0177533. doi: 10.1371/journal.pone.0177533 (PMC5464535; doi:10.1371/journal.pone.0177533)

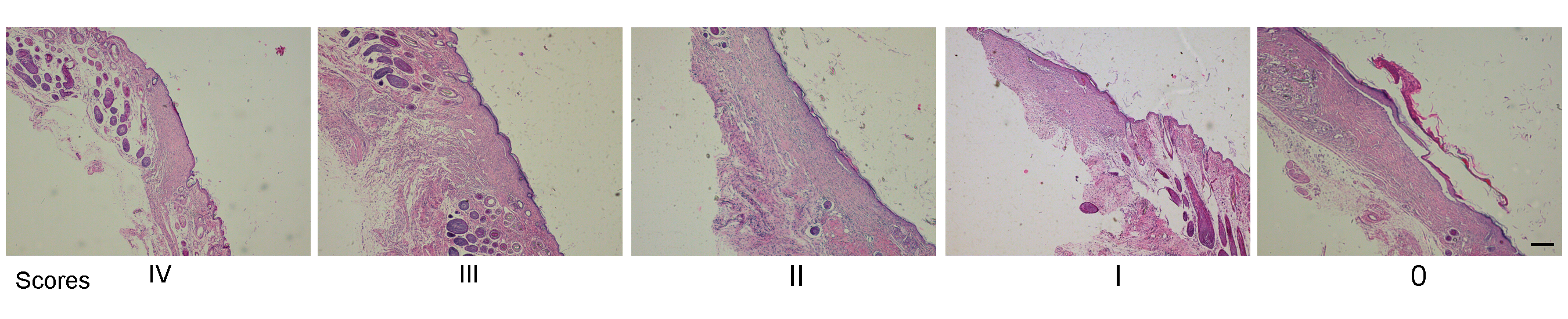

Supplement: S1 Fig — Representative images of haematoxylin-eosin staining of the dermo-epidermal junction integrity. Image J was used for estimating unbound area size. Scores: IV: 0.1–400 pixels (complete junction), III: 401–750 pixels, II: 751–1,200 pixels, I: 1,201–2,800 pixels and 0: > 2,801 (incomplete junction). Scale bar = 100 μm and applied to all images. (TIF) [file pone.0177533.s001.tif]

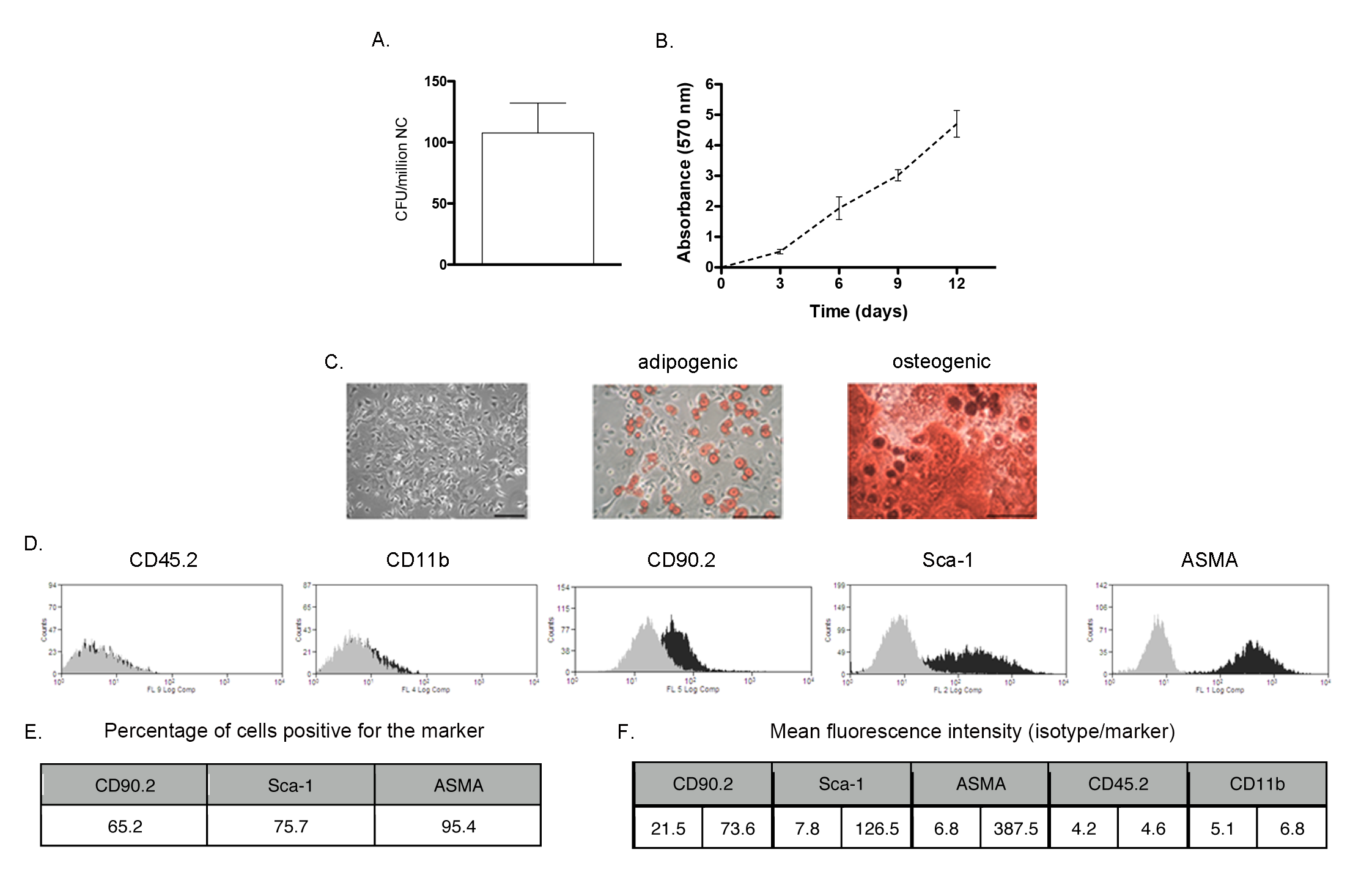

Supplement: S2 Fig — (A) Bone marrow abundance was determined by CFU assay. Data are mean ± s.e.m (n = 6). (B) Proliferation kinetics was evaluated by crystal violet staining (570 nm absorbance) over a period of 12 days. Data are mean ± s.e.m (n = 4). (C) mBM-MSCs differentiate to mesodermal lineages in vitro. Passage 2 mBM-MSCs were used for potential differentiation. Adipogenic differentiation: cells were cultured in adipogenic differentiation medium for 21 days. Oil Red O staining was performed to detect lipid accumulation. Osteogenic differentiation: cells were cultured in osteogenic differentiation medium for 21 days. Alizarin Red staining was performed to detect calcium accumulation. Scale bar 100 μm. (D) Flow cytometry analysis of mBM-MSC surface markers. mBM-MSCs expressed anti-CD90.2, anti-Sca-1, and anti-ASMA but not anti-CD45.2 and anti-CD11b. Representative images for 2 animals per group. (E) Statistical data of flow cytometry. Abbreviations: mBM-MSCs, mouse bone marrow-derived MSC and CFU, colony formation unit. (TIF) [file pone.0177533.s002.tif]

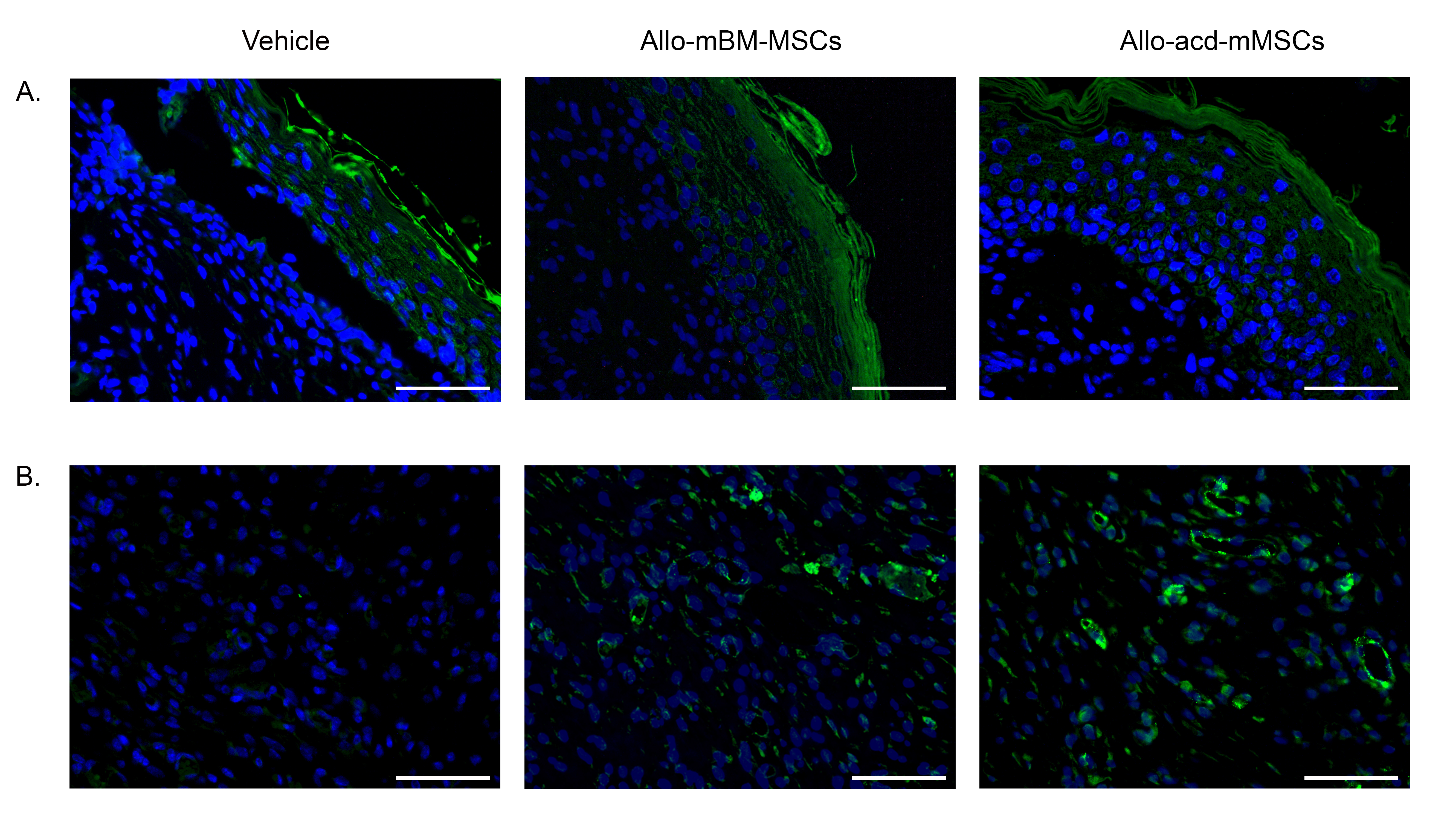

Supplement: S3 Fig — Representative images of cutaneous wound sections on day 16 in NOD mice treated with vehicle, allo-mBM-MSCs and allo-acd-mMSCs were immunostained with (A) an anti- pan-cytokeratin antibody (green) and (B) an anti-von Willebrand Factor (vWF) positive blood vessels (green), both primary antibodies were probed with Alexa Fluo488 secondary antibody and nuclei (blue) were counterstained with 4-6-diamidino-2-phenylindole (DAPI). Representative results of 4 animals per experimental groups. Scale bar 50 μm. Abbreviations: NOD, Non-Obese Diabetic; allo-mBM-MSCs, mouse bone marrow-derived allogeneic MSCs; allo-acd-mMSCs, mouse bone marrow acelullar derivatives allogeneic MSCs; vWF, von Willebrand Factor; DAPI 4-6-diamidino-2-phenylindole. (TIF) [file pone.0177533.s003.tif]

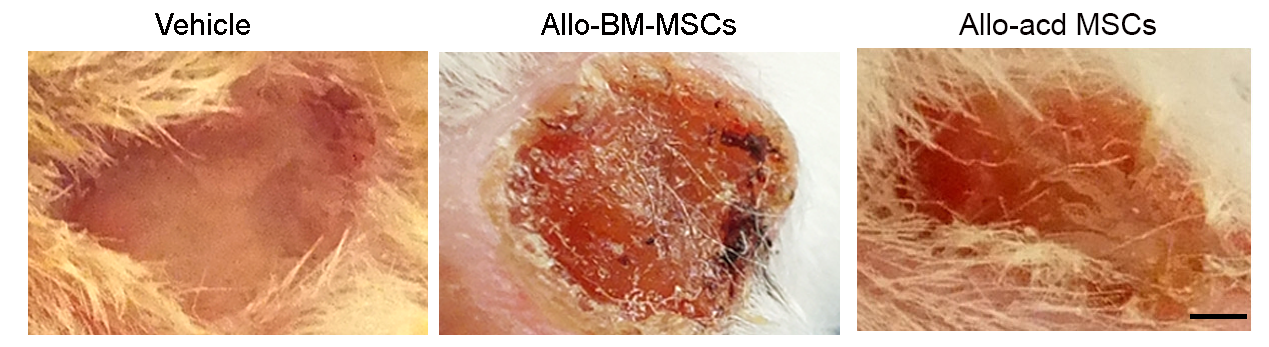

Supplement: S4 Fig — Invasion of granulation tissue is shown from the wound margins into the wound bed after 4 days of treatment. Allo-mBM-MSCs and allo-acd-mMSCs groups showed higher presence of tissue with the following features: wet, reddish, soft and granular in gross appearance compared to the vehicle group (only sparse granulation tissue was observed). Scale bar = 50 μm (applied to all images). Abbreviations: allo-mBM-MSCs, mouse bone marrow-derived allogeneic; acelullar derivatives, allo-acd-mMSCs. (TIF) [file pone.0177533.s004.tif]
